# Microtubule-Associated Protein 2 Expression in Canine Glioma

**DOI:** 10.3389/fvets.2019.00395

**Published:** 2019-11-15

**Authors:** Elena Alina Demeter, Chad Frank, Daniel R. Rissi, Brian F. Porter, Andrew D. Miller

**Affiliations:** ^1^Department of Biomedical Sciences, Section of Anatomic Pathology, Cornell University College of Veterinary Medicine, Ithaca, NY, United States; ^2^Department of Microbiology, Immunology, and Pathology, College of Veterinary Medicine and Biomedical Sciences, Colorado State University, Fort Collins, CO, United States; ^3^Athens Veterinary Diagnostic Laboratory and Department of Pathology, University of Georgia College of Veterinary Medicine, Athens, GA, United States; ^4^Department of Veterinary Pathobiology, College of Veterinary Medicine & Biomedical Sciences, Texas A&M University, College Station, TX, United States

**Keywords:** microtubule-associated protein 2, canine, glioma, oligodendroglioma, astrocytoma, immunohistochemistry

## Abstract

Canine glioma is considered a potential model for human glioma, with recent studies of occurrence, therapy, and reclassification supporting the value of the canine model. The current diagnosis of canine glioma is based on morphologic criteria and immunohistochemistry (IHC), including oligodendrocyte transcription factor 2 (Olig2), glial fibrillary acidic protein (GFAP), and 2′, 3′ cyclic nucleotide phosphatase (CNPase). Microtubule-associated protein 2 (MAP2) is a proven marker of human glioma and is used to complement the diagnosis and its specific immunoreactivity pattern contributes to the differentiation of astrocytomas from other glial tumors. The objective of this study was to evaluate whether canine gliomas express MAP2 and to explore differences in the pattern of immunolabeling between different gliomas. Seventy-eight cases of canine glioma were evaluated for MAP2 expression by immunohistochemistry. A glial origin was supported by Olig2 IHC in all cases. MAP2 immunolabeling was evaluated on a semi-quantitative basis, including the percentage of immunolabeled neoplastic cells, as well as the signal intensity, distribution, and pattern of immunolabeling. MAP2 was expressed in all cases, with significant correlation between diagnosis and signal intensity (*P* = 0.04). MAP2 immunolabeling distribution was dominated by diffuse (34/78; 44%), followed by patchy (20/78; 26%), multifocal to coalescing (16/78; 21%), and scattered (8/78; 10%). All oligodendrogliomas (53/53; 100%) and undefined gliomas (12/12; 100%) revealed a combination of perinuclear and cytoplasmic immunolabeling, and all but 3 astrocytomas had a combination of perinuclear and cytoplasmic processes immunolabeling (10/13; 77%). Significant correlation between immunolabeling pattern and diagnosis was obtained (*P* = 0.001). The study demonstrates that MAP2 is expressed in canine gliomas and the pattern of expression can also be applied to help distinguish astrocytomas from oligodendrogliomas and undefined gliomas.

## Introduction

Canine brain tumors are a potential naturally occurring model for their counterpart human disease and occur at a comparable incidence between the two species (1–5). Recent efforts to reclassify canine gliomas (6) have bridged the gap between canine and human glioma histopathology by employing a streamlined and more comprehensive tumor classification scheme. This reclassification has standardized the diagnoses and grading of canine glioma to oligodendroglioma, astrocytoma, or undefined representing either high- or low-grade neoplasms (6). Primary tumors of the central nervous system (CNS) in dogs are dominated by meningioma (~50%) (7), followed by gliomas (~40%) (8, 9), and choroid plexus tumors (~7%) (10, 11). Over 50% of gliomas occur in brachycephalic breeds, such as boxers, Boston terriers, and bulldogs. Even among the brachycephalic breeds, boxers, bulldogs, and Boston terriers have a reported higher incidence for astrocytomas in particular (12). The overall incidence of primary CNS tumors in dogs ranges from 3 to 5% of all neoplasms and is similar to their human counterparts (1, 13). The prognosis of dogs with primary CNS tumors is guarded to poor, and numerous cases are euthanized after the initial diagnosis. Therapeutic management of these cases, when elected, follows similar protocols as in humans, including surgical resection, radiation, and chemotherapy. Histologic characterization of the neoplasm is integral to deciding on the therapeutic course (14), but therapy is often attempted without fulfilling this step (15).

A variety of morphological and immunohistochemical (IHC) similarities between canine and human gliomas have been reported, particularly in astrocytomas (16–19). Olig2, a transcription factor, consistently labels canine glioma (20) with additional differentiation aided by expression of CNPase, for oligodendroglioma, and GFAP, for astrocytoma (6, 21). Furthermore, expression of growth factors and receptors, such as VEGF, EGFR-1, and PDGFR alpha, which correlate with higher grades for astrocytomas in humans, are also documented in dogs (18). In human neuropathology, molecular techniques are the norm for determining the diagnosis and prognosis; however, such techniques are not yet widely used in the veterinary field. Recent studies do indicate a wide variety of cell signaling abnormalities in canine glioma, many similar to those described in human glioma (22).

The cellular origin of gliomas is still a heavily debated topic (23, 24). Current theories are focused on neural stem cells (23); however, there is strong evidence that mature astrocytes can dedifferentiate and regain immature glial properties and be a source of neoplastic transformation (25, 26). While the cell of origin remains unclear in the dog, canine oligodendrogliomas do express a number of immunohistochemical markers of oligodendrocyte precursor cells including SOX10 and NG2, supporting a role for altered progenitor cells in the pathogenesis (27).

Microtubule-associated proteins (MAPs) are a group of cytoskeletal proteins of the MAP2/Tau family (28) that regulate homeostasis of microtubules. MAP2 binds to and increases the rigidity of the microtubules. Previous research has indicated that MAP2 plays an important role in forming dendritic protrusions in astrocytes and oligodendrocytes (28, 29). As cytoskeletal microtubules are essential for cell migration, interrupting microtubular dynamics would offer the potential of altering the infiltrative nature of glioma (30). MAP2 is found in two main forms, low molecular weight (MAP2c and MAP2d) and high molecular weight (MAP2a and MAP2b) protein (28). In the adult brain, MAP2a, 2b, and 2c are confined to neurons whereas MAP2d is associated with oligodendroglial precursors (31). The purpose of this study was to evaluate the expression of MAP2 in canine gliomas and determine if expression differences exist amongst oligodendroglioma, astrocytoma, and undefined gliomas.

## Methods

### Cases

A total of 78 canine gliomas were included in this study. Cases were retrieved from the pathology archives of Cornell University College of Veterinary Medicine, New York State Animal Health Diagnostic Center; University of Georgia College of Veterinary Medicine, Athens Diagnostic Laboratory; Colorado State University College of Veterinary Medicine; and Texas A&M College of Veterinary Medicine. All cases were fixed in 10% neutral buffered formalin, embedded in paraffin, sectioned at 5 μm, and stained with hematoxylin and eosin. All cases were classified and graded according to Koehler et al. (6).

### Immunohistochemistry Protocols and Evaluation

Using an online sequence alignment tool (https://blast.ncbi.nlm.nih.gov/Blast.cgi), the MAP2 sequences for the dog were compared to sequences for rat and human and sequence homology indicated that an antibody for MAP2 that was derived in a rat would be predicted to label canine tissue. IHC for MAP2 (Sigma-Aldrich, St. Louis, MO; catalog number M4403) and Olig2 (Abcam Cambridge, MA, catalog number EPR2673) were performed using the Leica BOND Max IHC Staining System (Leica Systems, Buffalo Grove, IL). Pretreatment with heat-induced antigen retrieval was performed for 30 min using Tris/EDTA pH 9 (Bond Epitope Retrieval Solution 2, Leica, catalog #AR9640) for MAP2 and Olig2. Endogenous peroxidase activity was blocked with a 3% peroxide solution for 5 min (Leica, catalog #DS9800). The antibodies were diluted at 1:1,000 for 60 min (MAP2) and 1:200 for 60 min (Olig2). Biotin-free PowerVision poly-polymeric horseradish peroxidase anti-mouse (MAP2) (Leica, catalog #PV6114) or anti-rabbit (Olig2) (Leica, catalog #PV6119) IgG reagent was then applied to the slides for 30 min, followed by incubation with Bond Polymer Refine Detection for 10 min (Leica, catalog #DS9800). Tissues were developed with 3,3-diaminobenzidine (DAB) (Leica, catalog #DS9800) for 10 min. The slides were counterstained with hematoxylin (Leica, catalog #DS9800) for 5 min, dehydrated, cleared, and mounted. A tissue microarray composed of a number of canine tissues including kidney, lung, liver, spleen, brain, heart, small intestine, testis, lymph node, and pancreas was used to determine the specificity of the antibody to canine nervous tissue. In the tissue microarray, labeling was consistently present in the brain sections. Positive controls consisted of normal canine brain where MAP2 immunolabeling is found in the cytoplasm of neurons. Negative controls consisted of an isotype-matched antibody.

The degree of signal intensity was measured on a semi-objective scale of 0–3 according to the following criteria: 0 = no labeling; 1 = less than 25% of neoplastic cells with immunolabeling; 2 = 25–75% of neoplastic cells with immunolabeling; 3 = more than 75% of neoplastic cells with immunolabeling. Percentage of positive cells were determined based on the overall immunoreaction within the entirety of the neoplasm. The overall strength of the signal intensity was scored based on reasonable staining differences from 0 to 3, with 0 corresponding to no detectable signal, and 1, 2, and 3 corresponding to low, intermediate, and strongest signal intensity, respectively. The distribution of the immunolabeling within the neoplasm was evaluated as diffuse (throughout the neoplasm), multifocal to coalescing (large areas of immunolabeling within the mass that are confluent), patchy (small clusters of neoplastic cells with immunolabeling), and scattered (mostly individualized cells with immunolabeling). The cellular pattern of immunolabeling was also evaluated as nuclear (Nc), perinuclear (PNc), cytoplasmic (Ct), cytoplasmic processes (CtP), or any combinations of these. The IHC immunolabeling was scored independently by EAD and by a board certified veterinary pathologist (ADM). Intensity scores were then determined for each case through a combined review of EAD and ADM's original scoring. Olig2 expression was evaluated for percentage of neoplastic cells with immunolabeling based on the same scoring system employed for MAP2. All data were statistically analyzed using JMP™ software (www.jmp.com).

## Results

**Figure 1:**
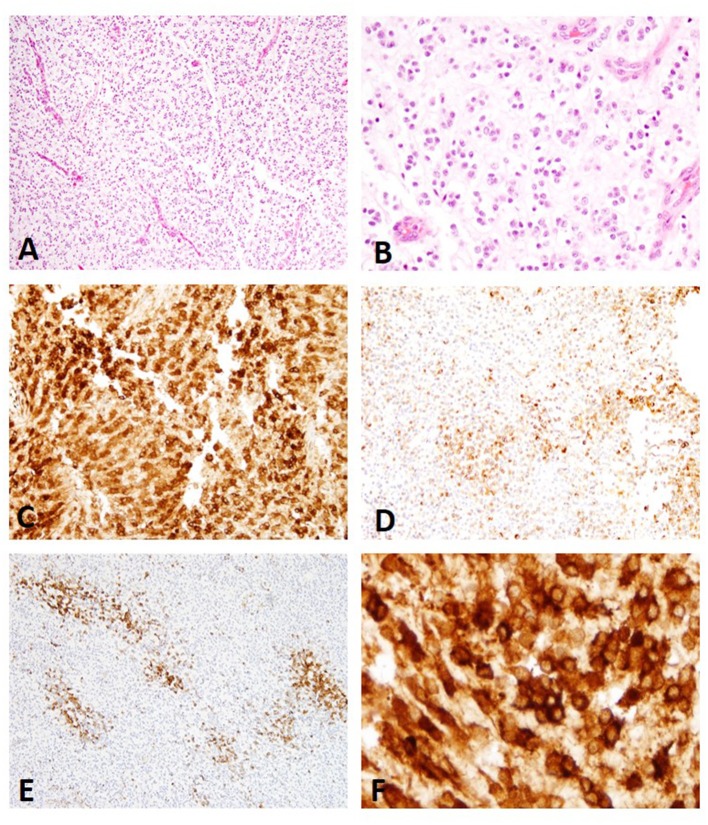
**(A)** Canine, Oligodendroglioma. Sheets of neoplastic oligodendrocytes with nuclear rowing and sparse stroma with branching capillaries (Hematoxylin and eosin (H&E) stain). **(B)** Canine, Oligodendroglioma. Round nuclei with coarse chromatin (H&E stain). **(C)** Canine, Oligodendroglioma. Diffuse immunolabeling with strong signal intensity (score 3) (Immunohistochemistry (IHC); MAP2). **(D)** Canine, Oligodendroglioma. Multifocal to coalescing distribution with moderate signal intensity (score 2) (IHC; MAP2). **(E)** Canine, Oligodendroglioma. Multifocal patchy immunolabeling with moderate signal intensity (score 2) (IHC; MAP2). **(F)** Canine Oligodendroglioma. Perinuclear and cytoplasmic immunolabeling pattern present in all cases of oligodendroglioma (IHC; MAP2).

**Figure 2:**
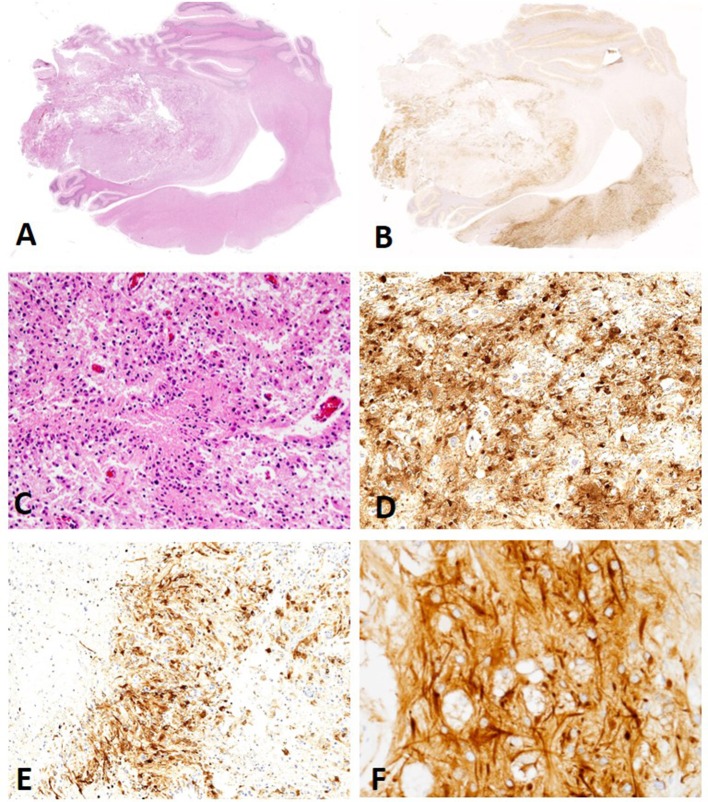
**(A)** Canine, Astrocytoma. Low-magnification of an astrocytoma (Hematoxylin and eosin (H&E) stain). **(B)** Canine, Astrocytoma. Low-magnification of MAP2 immunolabeling in an astrocytoma revealing multifocal to coalescing distribution with score 2 signal intensity (Immunohistochemistry (IHC); MAP2). **(C)** Canine, Astrocytoma. Low degree of cellular density, pleomorphism, and angular nuclei. (H&E stain). **(D)** Canine, Astrocytoma. Diffuse distribution of MAP2 immunolabeled cells in a case scored as a 3 for percent of neoplastic cells with immunolabeling (IHC; MAP2). **(E)** Canine, Astrocytoma. Patchy, multifocal distribution of MAP2 immunolabeled neoplastic cells (IHC; MAP2). **(F)** Canine, Astrocytoma. Immunolabeling of cytoplasmic processes (CtP) and perinuclear (PNc), a pattern noted in the majority of canine astrocytomas (IHC; MAP2.).

**Figure 3:**
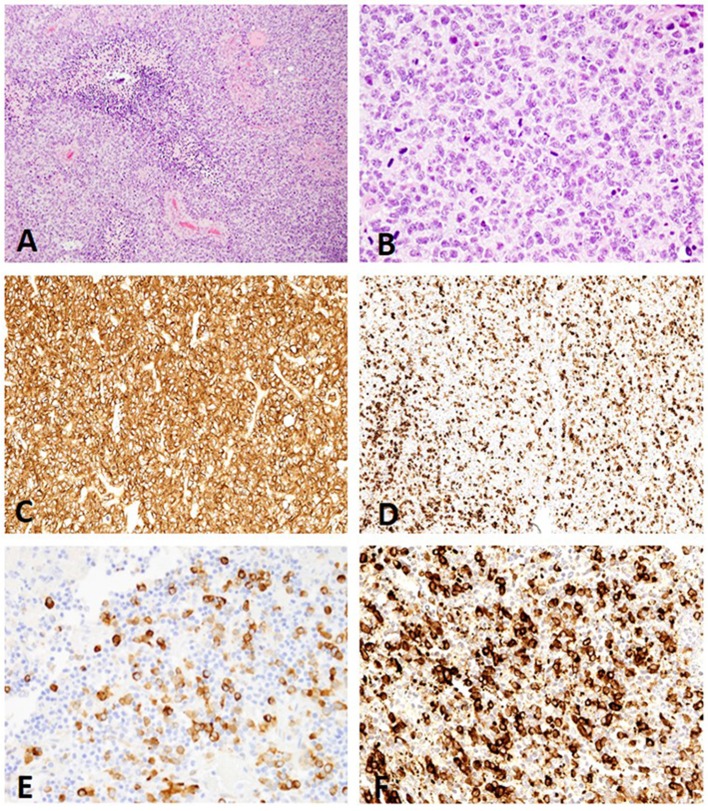
**(A)** Canine, Undefined glioma. Biphasic or biphenotypic morphology with palisading neoplastic cells around areas of necrosis. (Hematoxylin and eosin (H&E) stain). **(B)** Canine, Undefined glioma. Combination of nuclear rowing, pleomorphism, and high mitotic count (H&E stain). **(C)** Canine, Undefined glioma. MAP2 immunolabeling of canine undefined glioma revealing diffuse distribution with a score 3 signal intensity (Immunohistochemistry (IHC); MAP2). **(D)** Canine, Undefined glioma. Scattered distribution with a score 2 signal intensity (IHC; MAP2). **(E)** Canine, Undefined glioma. Scattered distribution with a score 1 signal intensity (IHC; MAP2). **(F)** Canine, Undefined glioma. The immunolabeling pattern, perinuclear (PNc) and cytoplasmic (Ct), noted in all undefined gliomas (IHC; MAP2).

The 78 cases included 34 different dog breeds, dominated by boxer (16/78; 21%), and followed by American bulldog (5/78; 6%), Boston terrier (4/78; 5%), and French bulldog (3/78; 4%), with individual cases corresponding to other breeds (Table 1). Spayed females made up 45% of all cases (35/78), followed by male castrated (36%; 28/78), male intact (8%; 6/78), and female intact (4%; 3/78), with 4/78 cases (5%) recorded as male not otherwise specified (NOS) and 2/78 (3%) cases with unknown sex. Ages ranged from 1 to 15 with an average of 7.6 years old and a median of 8 years old. The anatomic location of the neoplasm was dominated by forebrain NOS (20/78; 26%), followed by right forebrain (16/78; 21%), left forebrain (14/78; 18%), and cerebellum (5/78; 6%), with other anatomic locations represented in lower numbers (Table 1). The 78 glioma cases consisted of oligodendroglioma (Figure 1), astrocytoma (Figure 2), and undefined glioma (Figure 3). Of each diagnostic category, there were 53 oligodendrogliomas (68%; of which 32 were high-grade and 21 were low-grade; Figures 1A,B); 13 astrocytomas (17%; of which 2 were high-grade and 11 were low-grade; Figures 2A,C); and 12 undefined gliomas (15%; of which 7 were high-grade and 5 were low-grade; Figures 3A,B).

**Table 1:** Canine glioma cases included in the study with demographic data.

**Case**	**Diagnosis**	**Grade**	**Breed**	**Age (years)**	**M/F/MC/FS**	**Location**
1	Oligodendroglioma	HG	Cocker Spaniel	8	MC	Left forebrain
2	Oligodendroglioma	HG	Boston Terrier	9	FS	Left forebrain
3	Oligodendroglioma	HG	Mixed	nr	nr	Forebrain, NOS
4	Oligodendroglioma	HG	Mixed	5	MC	Spinal cord, NOS
5	Oligodendroglioma	HG	Boxer	2	FS	Intraventricular
6	Oligodendroglioma	HG	Boxer	1	FS	Spinal cord, thoracolumbar
7	Oligodendroglioma	HG	Mixed breed	10	MC	Forebrain, NOS
8	Oligodendroglioma	HG	Boxer	4	FS	Right forebrain
9	Oligodendroglioma	HG	Boxer	5	MC	Left forebrain
10	Oligodendroglioma	HG	Boston Terrier	8	FS	Left forebrain
11	Oligodendroglioma	HG	Munsterlander	13	FS	Right forebrain
12	Oligodendroglioma	HG	Staffordshire terrier	11	FS	Left forebrain
13	Oligodendroglioma	HG	Labradoodle	11	FS	Right forebrain
14	Oligodendroglioma	HG	Labrador retriever cross	11	MC	Forebrain, left and right
15	Oligodendroglioma	HG	Silky terrier	15	MC	Forebrain, left and right
16	Oligodendroglioma	HG	French bulldog	8	FS	Forebrain, left and right
17	Oligodendroglioma	HG	Poodle	8	FS	Right forebrain
18	Oligodendroglioma	HG	Mixed breed	8	FS	Left forebrain
19	Oligodendroglioma	HG	Jack Russell Terrier	5	FS	Forebrain, NOS
20	Oligodendroglioma	HG	Boxer	7	FS	Midbrain
21	Oligodendroglioma	HG	Boxer	13	MC	Forebrain, NOS
22	Oligodendroglioma	HG	Labrador retriever	2	MC	Forebrain, NOS
23	Oligodendroglioma	HG	American bulldog	10	FS	Forebrain, NOS
24	Oligodendroglioma	HG	Boston terrier	8	FS	Left forebrain
25	Oligodendroglioma	HG	German shepherd	9	MC	Forebrain, NOS
26	Oligodendroglioma	HG	Boxer	13	MC	Right forebrain
27	Oligodendroglioma	HG	French Bulldog	7	M	Right forebrain
28	Oligodendroglioma	HG	Boxer	5	MC	Left forebrain
29	Oligodendroglioma	HG	Mixed breed	2	MC	Left forebrain
30	Oligodendroglioma	HG	Labrador retriever	8	FS	Right forebrain
31	Oligodendroglioma	HG	Boxer	8	MI	Midbrain
32	Oligodendroglioma	HG	West highland white terrer	5	FS	Spinal cord, cervical
33	Oligodendroglioma	LG	Boxer	9	MI	Right forebrain
34	Oligodendroglioma	LG	English bulldog	3	FS	Right forebrain
35	Oligodendroglioma	LG	Boxer	1	FS	Spinal cord, thoracolumbar
36	Oligodendroglioma	LG	American bulldog	7	MC	Forebrain, NOS
37	Oligodendroglioma	LG	Mixed breed	10	MC	Right forebrain
38	Oligodendroglioma	LG	Borzoi	7	FS	Midbrain
39	Oligodendroglioma	LG	Bulldog, NOS	6	MC	Forebrain, NOS
40	Oligodendroglioma	LG	French Bulldog	5	MC	Midbrain
41	Oligodendroglioma	LG	Staffordshire terrier	7	FS	Forebrain, NOS
42	Oligodendroglioma	LG	Boxer	13	MI	Forebrain, NOS
43	Oligodendroglioma	LG	Wirehaired fox terrier	11	MI	Forebrain, NOS
44	Oligodendroglioma	LG	Boston terrier	10	FS	Cerebellum
45	Oligodendroglioma	LG	Boxer	2	FS	Intraventricular
46	Oligodendroglioma	LG	Staffordshire terrier	7	FS	Forebrain, NOS
47	Oligodendroglioma	LG	Staffordshire terrier	6	FI	Midbrain
48	Oligodendroglioma	LG	Cock-a-poo	4	FS	Left forebrain
49	Oligodendroglioma	LG	Boxer	13	MI	Forebrain, NOS
50	Oligodendroglioma	LG	nr	nr	nr	Forebrain, NOS
51	Oligodendroglioma	LG	Boxer	13	MC	Forebrain, NOS
52	Oligodendroglioma	LG	American bulldog	7	MC	Forebrain, NOS
53	Oligodendroglioma	LG	Boxer	9	MC	Right forebrain
54	Astrocytoma	LG	Mixed breed	10	MC	Forebrain, NOS
55	Astrocytoma	LG	Australian heeler	14	M	Cerebellum
56	Astrocytoma	LG	Australian cattle dog	4	FS	Spinal cord, NOS
57	Astrocytoma	LG	Mixed breed	7	MC	Left forebrain
58	Astrocytoma	LG	American Eskimo	12	FS	Right forebrain
58	Astrocytoma	LG	Mixed breed	13	FS	Right forebrain
60	Astrocytoma	LG	Mastiff, NOS	7	FS	Right forebrain
61	Astrocytoma	LG	Italian greyhound	10	FS	Right forebrain
62	Astrocytoma	HG	English bulldog	4	FS	Left forebrain
63	Astrocytoma	HG	Staffordshire terrier	7	FS	Cerebellum
64	Astrocytoma	LG	French bulldog	8	MC	Cerebellum
65	Astrocytoma	LG	Mixed breed	10	MC	Cerebellopontine angle
66	Astrocytoma	LG	Jack Russell Terrier	8	FS	Cerebellum
67	Undefined glioma	LG	American bulldog	3	FS	Midbrain
68	Undefined glioma	LG	Boston terrier	5	MC	Right and left forebrain
69	Undefined glioma	LG	Standard poodle	8	MC	Midbrain
70	Undefined glioma	LG	Bull Mastiff	Adult, NOS	MI	Forebrain, NOS
71	Undefined glioma	LG	French bulldog	11	FS	Left forebrain
72	Undefined glioma	HG	Staffordshire terrier	9	MC	Spinal cord, NOS
73	Undefined glioma	HG	Boxer cross	9	MC	Right forebrain
74	Undefined glioma	HG	Staffordshire terrier	5	MC	Right and left forebrain
75	Undefined glioma	HG	Border Terrier	13	FI	Spinal cord, NOS
76	Undefined glioma	HG	Boston terrier	7	FI	Left forebrain
77	Undefined glioma	HG	American bulldog	1	M	Forebrain, NOS
78	Undefined glioma	HG	Golden retriever	2	M	Right and left forebrain

*nr, not reported*.

*MAP2 immunolabeling percentage:* Equal numbers of gliomas were scored 3 (30/78; 38.5%) and 2 (30/78; 38.5%), while fewer scored 1 (18/78; 23%). There were no cases with 0 scoring on MAP2 scoring, allowing application of the null hypothesis (32), that reveals a 0.037% chance that a 0 score can happen with a canine glioma.

*MAP2 immunolabeling signal intensity:* Most cases had score 3 signal intensity (30/78; 38.5%), followed by 2 (28/78; 36%) and 1 (20/78; 26%). Significant correlation between type of glioma and signal intensity was found, regardless of the grade (*P* = 0.04).

*MAP2 immunolabeling distribution:* The distribution was dominated by diffuse immunolabeling (34/78; 44%), followed by patchy (20/78; 26%), multifocal to coalescing (16/78; 21%), and scattered (8/78; 10%).

*MAP2 cellular pattern of immunolabeling:* All oligodendrogliomas (53/53; 100%) and undefined gliomas (12/12; 100%) had a combination of PNc and Ct staining (Figures 1C–F, 3C–F). Three astrocytomas (3/13; 23%) had similar PNc and Ct staining; however, the remaining astrocytoma cases had a combination of PNc and CtP staining (10/13; 77%; Figures 2B,D–F). A significant correlation between staining pattern and diagnosis was obtained (*P* = 0.001). Specifically astrocytomas were more likely to stain with a combination of PNc and CtP, and oligodendrogliomas and undefined gliomas were more likely to stain with a PNc and Ct pattern (Supplemental Figures 1, 2).

*MAP2 scoring and neoplasm grading (low vs. high-grade) comparison:* Most high-grade gliomas (18/41; 44%) were assigned a score of 2 when analyzing the MAP2 immunolabeling percentage, followed by 3 (16/41; 39%), and 1 (7/41; 17%). A more even distribution was noted in the low-grade glioma group with most tumors assigned a score 3 (14/37; 38%), followed by score 2 (12/37; 32%), and score 1 (11/37; 30%). The majority of low-grade tumors had a PNc and Ct staining pattern (78%), and the remaining 21% had PNc and CtP staining. Ninety-five percent of the high-grade tumors had a PNc and Ct staining pattern, and only 5% had a PNc and CtP pattern. Overall, no correlation could be achieved between tumor grade and MAP2 signal intensity score or immunolabeling pattern. Olig2 expression was only used to score the percentage of neoplastic cells with immunolabeling. The majority of cases had a score of 3 (45/78; 58%), followed by 2 (28/78; 36%), and 1 (5/78; 6%) for Olig2 labeling.

## Discussion

The classification of the gliomas included in this study was based on Koehler et al. (6). Based on these new criteria, glioma diagnoses are divided into oligodendroglioma, astrocytoma, and undefined, where the latter contains a roughly equal distribution of the two former glioma subtypes. Criteria considered for oligodendroglioma are: round nuclei, coarse chromatin, nuclear rowing, artifactual loss of cytoplasm, branching capillaries, and pseudo-rosettes. For astrocytoma, the criteria are: angular nuclei, open chromatin, pleomorphism, and a lower degree of cellular density than oligodendroglioma. The distribution of glioma subtypes in this study is similar to previous findings (6), with most cases being oligodendroglioma. Determining the exact prevalence and anatomic distribution of these tumors is challenging in veterinary medicine, due to data heterogeneity and inconsistent diagnostic criteria. While sample size is a limiting factor for strong statistical correlations, boxers were overrepresented in this study, similar to other previous reports (2, 10, 11). A susceptibility locus on canine chromosome 26 has been associated with a predilection for glioma in brachycephalic breeds (2, 22), but the routine molecular analyses necessary to confirm this predilection are nascent in veterinary medicine. The average age of the dogs in our study was 7.6 years, similar with previous reports, with 1 year old and 15 years old the minimum and maximum age, respectively.

Numerous monoclonal and polyclonal antibodies have been applied in the diagnosis of canine CNS tumors (8, 33), which allows for a more accurate correlation with their human counterparts. MAP2 is a proven marker in human gliomas, consistently used for diagnostic and grading purposes (31, 34–36), but its potential application in canine gliomas had not been explored to date. While sample size is a limiting factor, all cases included in this study had at least some degree of immunolabeling for MAP2. Furthermore, the majority of gliomas were scored as either a score 2 (38.5%) or a score 3 (38.5%), with significant correlation between diagnosis of glioma and immunolabel intensity (*P* = 0.04). MAP2 expression in human glioma reveals different immunolabeling patterns between oligodendroglioma (i.e., sparse processes, that in our study corresponds to lack of CtP immunolabeling and prominent PNc and Ct immunolabeling) and astrocytoma (i.e., densely ramified astrocytic elements, that correspond to predominately CtP staining in majority of the canine astrocytoma in this study) (37). All oligodendrogliomas (53/53; 100%) and undefined gliomas (12/12; 100%) in this study had a combination of PNc and Ct immunolabeling. Except for three astrocytomas (3/13; 23%) that had a combination of PNc and Ct immunolabeling, the majority of astrocytomas had a combination of PNc and CtP staining (10/13; 77%), with robust highlighting of cytoplasmic processes. Significant correlation between staining pattern and diagnosis was obtained (*P* = 0.001).

Based on these findings, MAP2 appears to be a suitable diagnostic immunohistochemical marker for canine glioma, with strong potential of aiding in the diagnosis of astrocytoma based on the pattern of immunolabeling. However, the number of cases exhibiting multifocal to coalescing (16/78; 20.5%) and patchy immunolabeling (20/78; 26%) illustrate the heterogeneity present in these tumors and raise a concern that the use of tissue microarray for immunohistochemical analysis in canine glioma may yield results that are not entirely representative of the totality of the neoplasm (33, 38). The three cases of astrocytoma with PNc and Ct staining were all from one location (the cerebellar white matter) and had an unusual pattern composed of sheets of gemistocyte-like morphology. Whether this corresponds to a specific subtype of astrocytoma remains to be determined; however, tumors of this morphology have been described at this location in the dog (6). No correlation could be demonstrated between tumor grade and MAP2 immunolabeling intensity score, and this may be due to the small size and heterogeneity of the sample. The distribution of the data is summarized in Supplemental Figures 1, 2.

The overall statistical significance is difficult to interpret due to a limited and somewhat heterogeneous sample size; however, the IHC results presented herein suggest that MAP2 can be a valuable marker in the evaluation of canine glioma. Furthermore, the staining pattern obtained in this study is similar with findings in human gliomas (37), making this marker a tool for better understanding protein expression in canine glioma, but also for differentiating astrocytomas from oligodendrogliomas and undefined gliomas. Future directions of study should be focused on understanding the role that neuronal differentiation has in the pathogenesis of canine glioma.

## Data Availability Statement

All datasets generated for this study are included in the article/Supplementary Material.

## Ethics Statement

This study protocol did not require ethics approval according to local legislation and national guidelines.

## Author Contributions

This study was conceived by AM and ED. Case acquisition and interpretation was done by AM, ED, BP, CF, and DR. Experiments were performed by AM and ED. Data interpretation was done by AM and ED. Paper writing and editing was done by AM, ED, BP, CF, and DR.

### Conflict of Interest

The authors declare that the research was conducted in the absence of any commercial or financial relationships that could be construed as a potential conflict of interest.

## Supplementary Material

Supplemental Figure 1 Distribution of all data evaluated by JMP software.

Supplemental Figure 2 Distribution of diagnoses by the criteria considered for MAP2 immunolabeling evaluation (scoring, intensity, distribution, pattern) achieved with JMP software.
