# Supplementary figures and images for: Microtubule-Associated Protein 2 Expression in Canine Glioma
Source: Front Vet Sci. 2019 Nov 15;6:395. doi: 10.3389/fvets.2019.00395 (PMC6872496; doi:10.3389/fvets.2019.00395)

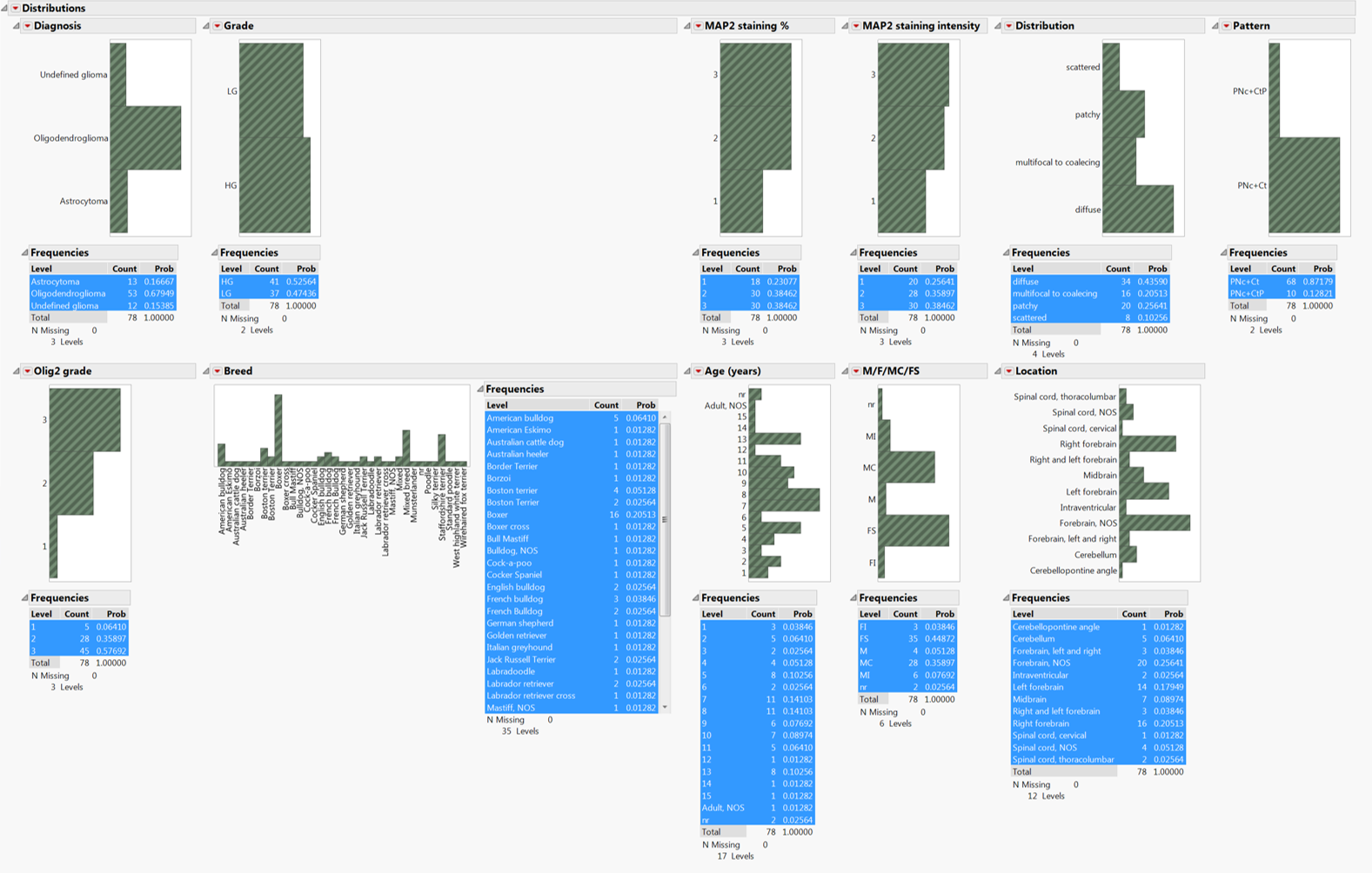

Supplement: Supplemental Figure 1 — Distribution of all data evaluated by JMP software. [file Image_1.TIF]

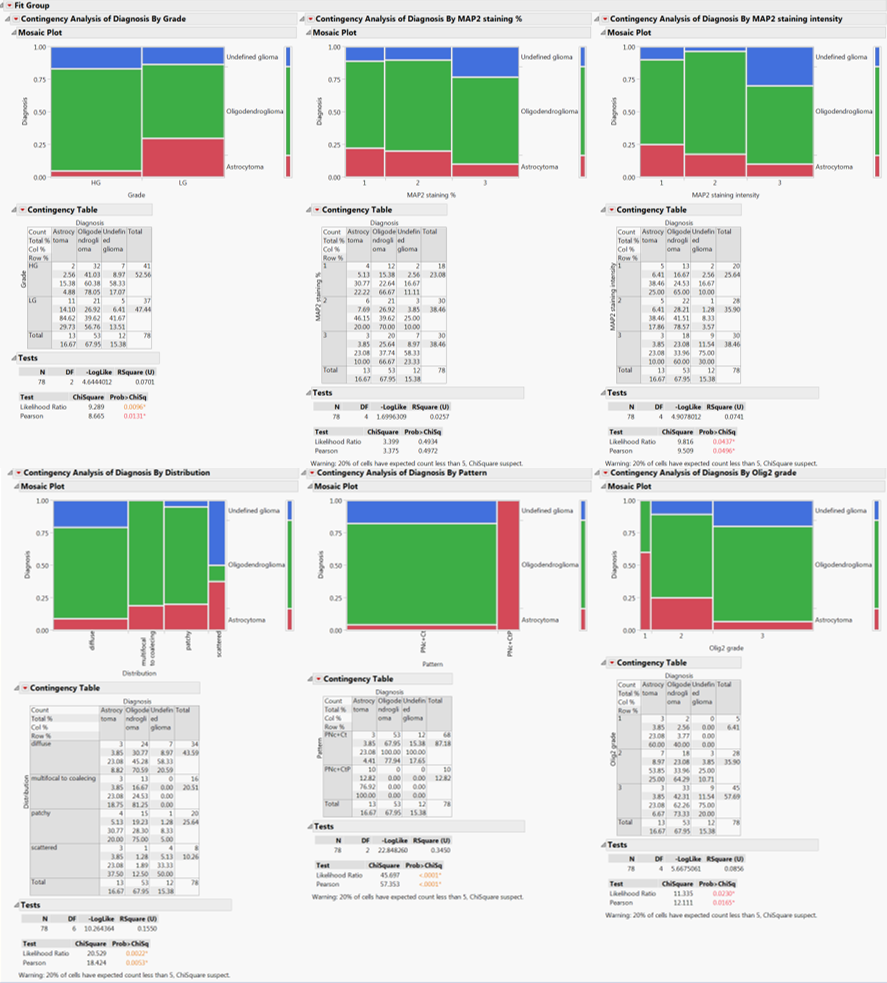

Supplement: Supplemental Figure 2 — Distribution of diagnoses by the criteria considered for MAP2 immunolabeling evaluation (scoring, intensity, distribution, pattern) achieved with JMP software. [file Image_2.TIF]
